# Population attributable fractions of depression and anxiety among Aboriginal and Torres Strait Islander peoples: a population-based study

**DOI:** 10.1016/j.lanwpc.2024.101203

**Published:** 2024-09-20

**Authors:** Subash Thapa, Kedir Y. Ahmed, Santosh Giri, Anayochukwu E. Anyasodor, M. Mamun Huda, Peter Gibbs, Shakeel Mahmood, Feleke H. Astawesegn, Jamie Newman, Allen G. Ross

**Affiliations:** a Rural Health Research Institute (RHRI), Charles Sturt University, Orange, NSW, 2800, Australia; b Regional Enterprise Development Institute (REDI.E), Dubbo, NSW, 2830, Australia; c Orange Aboriginal Medical Service (OAMS), Orange, NSW, 2800, Australia

**Keywords:** Aboriginal and Torres Strait Islander peoples, Anxiety disorder, Depression, Discrimination, Intergenerational trauma, Population attributable fraction, Stolen generation

## Abstract

**Background:**

Aboriginal and Torres Strait Islander peoples face an increased risk of common mental disorders, which may be associated with underlying socio-economic challenges, racism, and discrimination. This is the first study to calculate the population attributable fractions (PAFs) for depression and anxiety attributed to potentially modifiable risk factors such as health behaviour, social and cultural characteristics, and past adverse events among Aboriginal and Torres Strait Islander peoples aged ≥15 years.

**Methods:**

This cross-sectional study examined the 2018–19 National Aboriginal and Torres Strait Islander Health Survey conducted by the Australian Bureau of Statistics. Logistic regression models were used to compute odds ratios (ORs). PAFs adjusted for communality were calculated using adjusted ORs and prevalence estimates for each risk factor.

**Findings:**

This study included a weighted sample of 5362 individuals, with a mean age of 40.8 years (SD = ±17.2). Personal income below the national average (PAF = 13.4%; 95% CI: 12.4, 14.5), severed access to Indigenous cultural affiliations (PAF = 12.8%; 95% CI: 11.8, 13.8), central obesity (PAF = 7.2%; 95% CI: 6.4, 8.0), daily smoking (PAF = 5.9%; 95% CI: 5.2, 6.7) and severed access to Indigenous knowledge (PAF = 5.2%; 95% CI: 4.5, 5.8) were associated with 45% of depression cases. Personal income below the national average (PAF = 10.7%; 95% CI: 9.8, 11.7), limited access to Aboriginal Community Controlled Health Services (PAF = 10.6%; 95% CI: 9.7, 11.6), central obesity (PAF = 7.1%; 95% CI: 6.3, 7.9), severed access to Indigenous knowledge (PAF = 5.7%; 95% CI: 4.9, 6.4) and the experience of discrimination in the last 12 months (PAF = 4.7%; 95% CI: 4.0, 5.3) were associated with 39% of anxiety cases.

**Interpretation:**

To reduce the burden of depression and anxiety disorder among Aboriginal and Torres Strait Islander peoples, addressing socio-economic and cultural harms that constrain healthy connections to people/kin, their rights, languages, land, and healthy food sources should be a priority.

**Funding:**

This work was funded by a grant from the Commonwealth of Australia, represented by the 10.13039/501100003921Department of Health and Aged Care (Grant Activity 4-DGEJZ1O/4-CW7UT14).

**Research in context**

**Evidence before this study**

Indigenous populations in Australia bear the highest burden of mental disorders, which are associated with poorer lifestyles, increased rates of chronic health conditions, suicidal ideation, and premature deaths. The increased burden of mental disorders among Aboriginal and Torres Strait Islander peoples is linked to the socio-economic challenges, racism, and discrimination they experienced across multiple generations. There is a lack of empirical evidence on the most important risk and resilience factors within broader social, cultural, historical, and political determinants of mental health and wellbeing among Indigenous populations.

**Added value of this study**

Our study utilized the data from 5362 individuals to investigate population attributable fractions (PAF) of depression and anxiety disorder, attributable to modifiable risk factors associated with health behaviour, social and cultural characteristics, and past adverse events. PAF allows us to estimate the proportion of these diseases attributable to these risk factors and the potential reduction in disease burden if the prevalence of these risk factors were hypothetically reduced to zero. Unlike Odds Ratios/Risk Ratios, PAF considers both the strength of the relationship between the risk factor and disease and the prevalence of the risk factor in the population. This enables us to prioritize interventions with the greatest potential for reducing the burden of depression and anxiety.

**Implications of all the available evidence**

Modifiable risk factors associated with 45% of depression cases include lower personal income, severed access to Indigenous cultural affiliation, central obesity, daily smoking, and severed access to Indigenous knowledge. Modifiable risk factors associated with 39% of anxiety cases include lower personal income, limited access to Aboriginal Community Controlled Health Services, central obesity, severed access to Indigenous knowledge, and experiencing discrimination in the last 12 months. The current policies and practices aimed at reducing anxiety disorder and depression among Aboriginal and Torres Strait Islander peoples need to prioritize addressing socio-economic challenges, racism, and discrimination that constrain healthy connections to people/kin, their rights, languages, land, and healthy food sources. The rising rates of co-occurring mental disorders and central obesity could be addressed using personalized interventions, such as social prescribing, within community and primary care settings. State and national governments should increase investment in implementing Indigenous mental health and wellbeing programs to foster Indigenous self-determination and leadership.

## Introduction

Mental disorders are one of the major causes of health-related burden and premature death among Australians, with depression and anxiety disorder being leading contributors to this burden.^1^ With the increasing burden of mental disorders nationally, Australia's total expenditure on mental health-related services also increased from $10.9 billion in 2017–18 to $12.2 billion in 2021–22, showing an average annual growth rate of 4%.^2^ Within the Australian population, Aboriginal and Torres Strait Islander peoples bear the highest burden of mental disorders, accounting for 23% (55,200 years) of the total years of healthy life lost in 2018 (12% in all Australians in 2022) and experiencing a 52% increase in hospitalization rates between 2009 and 2018 (increasing from 19 to 29 per 1000 hospitalizations).^3^

Among Aboriginal and Torres Strait Islander peoples, the trauma from historical events associated with colonization, such as forced removal of children from their families, loss of Indigenous lands, and decades of neglect and discrimination, has been passed down through generations.^4^^,^^5^ They face systemic racism and discrimination, as well as overlapping economic deprivation and compromised family dynamics.^6^^,^^7^ The impacts of intergenerational trauma, systemic racism, and discrimination drive inequality in mental health outcomes for Indigenous population groups, with certain sub-populations, including females and those residing in rural areas, facing a higher risk.^8^^,^^9^ Mental disorders are further associated with poorer lifestyles, increased rates of chronic health conditions, suicidal ideation, and premature deaths, contributing to 20% of the Indigenous health gap.^10^^,^^11^

Previous studies have discussed the increased burden of mental disorders among Aboriginal and Torres Strait Islander peoples as being linked to socio-economic, cultural, and political disadvantages they experienced across multiple generations.^4^^,^^7^^,^^12^, ^13^, ^14^, ^15^, ^16^ Some studies also highlighted the protective factors linked to Indigenous cultural identity and practices which appear to provide resilience even in the presence of structural disadvantages, such as poverty, crowded living conditions and racism.^4^^,^^17^^,^^18^ However, there is a dearth of empirical evidence on the most important risk and resilience factors within the broader social, cultural, historical, and political determinants of mental health and wellbeing among Aboriginal and Torres Strait Islander peoples.

Because social determinants of wellbeing including poverty and poorer living conditions, systemic racism, intergenerational trauma, and high incarceration rates can lead to severe and enduring effects, recovery can only be facilitated through culturally and contextually appropriate interventions grounded in the most current evidence.^4^ However, existing strength-based approaches targeting Aboriginal and Torres Strait Islander communities, including building trust, policies for equitable job conditions, and educational interventions tend to have a narrow focus on individual resilience (e.g., skills, attitudes, norms, practices) rather than on social and structural factors that contribute to the wellbeing on a broader spectrum.^19^, ^20^, ^21^, ^22^, ^23^ While evidence on effectiveness of culturally tailored mental health and wellbeing programs incorporating Indigenous culture (e.g., incorporating Elders’ teachings,^22^ Indigenous festivals^24^) and support groups^25^ (e.g., empowering Indigenous peoples^21^) mostly comes from small-scale pilot studies, limiting their applicability and effectiveness to a larger population size.

Prioritizing and effectively addressing depression and anxiety disorder within Aboriginal and Torres Strait Islander communities will be crucial for ‘Closing the Gap’ agenda.^26^ The Australian National Preventive Health Strategy 2021–2030 states to increase the investment on prevention and early intervention of common mental disorders and develop enhanced social and mental health systems throughout the country.^27^ Despite this, providing a comprehensive, culturally safe, stepped-care approach for Indigenous populations still remains a challenge due to resource constraints and lack of mental health workforce, particularly in regional and rural Australia.^16^^,^^28^ Lack of culturally safe strategies within current programs and services, as well as language and cultural barriers, geographical remoteness, and stigma inhibit access to mental health services for more than 200 culturally and linguistically diverse subpopulations.^29^^,^^30^

Given resource limitations, prioritizing interventions that target the most modifiable risk factors associated with depression and anxiety disorder among Aboriginal and Torres Strait Islander peoples offers a pragmatic approach. However, there is a lack of evidence on what risk and resilience factors among the multi-level determinants of mental health and wellbeing should be prioritized while designing policies and programs for Aboriginal and Torres Strait Islander peoples. Population attributable fractions (PAFs) estimates for anxiety and depression offers crucial insights for prioritizing interventions, allocating resources effectively, and designing tailored prevention strategies for Indigenous populations of Australia. By quantifying the impact of modifiable risk factors, such as socio-economic factors, health risk behaviours, cultural factors, and adverse experiences, the PAF informs targeted efforts aimed at reducing the burden of depression and anxiety disorder and improving the overall well-being of Indigenous communities.

This study calculated the PAFs of depression and anxiety disorder, attributable to modifiable risk factors associated with health behaviour, social and cultural characteristics, and past adverse events among Aboriginal and Torres Strait Islander peoples.

## Methods

### Study design and data sources

This cross-sectional study examined the 2018–19 National Aboriginal and Torres Strait Islander Health Survey (NATSIHS). The 2018–19 NATSIHS was designed to collect a wide range of information about the health and wellbeing of Aboriginal and Torres Strait Islander peoples including individuals' demographics, nutrition, social determinants of health, chronic diseases including mental health conditions, and experiences of harm.^31^ Funding for the survey was provided by the Australian Government Departments of Health and Prime Minister and Cabinet.

### Sampling procedures and sample size

The 2018–19 NATSIHS was conducted by the Australian Bureau of Statistics (ABS) between July 2018 and April 2019. The survey used different sampling strategies to select the study participants from community and non-community Indigenous populations. The community sample included a random selection of discrete Indigenous communities and associated outstations from the Dwelling Register for Aboriginal and Torres Strait Islander Communities. Discrete Indigenous communities or outstations include: (a) communities in urban areas where the title to a parcel of land has been transferred to an Indigenous organization, such as communities on former mission or reserve land in New South Wales and Queensland; (b) well-established Indigenous communities and outstations in remote areas; (c) Deed of Grant in Trust (DOGIT) communities and their outstations in Queensland, as well as the two shires of Aurukun and Mornington Island; and (d) communities on Indigenous pastoral properties/leases.^31^^,^^32^

The non-community sample involved multistage area sampling of private dwellings outside Indigenous communities. Mesh blocks with First Nations households from the 2016 census were identified. Mesh blocks are the smallest geographical units defined in the Australian Statistical Geography Standard (ASGS), typically containing 30–60 dwellings. Dwellings in each mesh block were randomly selected. In non-remote areas, up to two adults aged 18 years or older and one child aged 15–17 years were randomly selected from both the community and non-community samples, while in remote areas, up to one adult and one child aged 15–17 years were randomly selected.^31^ The ABS obtained informed consent from the study participants before the interviews and for the secondary use of their data.

### Outcome variables

The outcome variables were depression and anxiety disorder. These conditions were identified at the time of the survey based on whether the respondent had been diagnosed with depression or anxiety disorder by a health professional and which had lasted at least six months, or the respondent expected to persist for six months or more.^31^^,^^33^ Classification of mental health conditions in the NATSIHS adhered to the International Statistical Classification of Diseases and Related Health Problems, 10th Revision (ICD-10).^31^ Development and cultural adaptation of the tools to measure the outcomes variables were informed through advice from an expert advisory panel, and the questions were tested in non-remote and remote areas of Australia. For the logistic regression models, the outcome variables were categorized as either “depressed” or “had anxiety” (coded as ‘1’) and “not depressed” or “no anxiety” (coded as ‘0’).

### Explanatory variables

The explanatory variables were broadly categorised into four groups: socioeconomic factors, health risk factors, cultural factors, and adverse experiences in the past. The socioeconomic factors included personal income, highest educational attainment, and place of residence. The health risk factors included daily smoking, excessive alcohol drinking (>21 units per week), physical inactivity, and central obesity. The cultural factors included affiliation to an Indigenous tribe or language group, main language spoken at home, level of Indigenous knowledge, and access to Aboriginal Community Controlled Health Services (ACCHS). The adverse experiences included discrimination (unfair treatment) because of indigenous backgrounds (e.g., being called names, hearing racial comments, being ignored, not trusted, unfairly arrested, humiliated, having objects thrown at them), physical harm in the last 12 months, and ever removed from one's natural family. Table 1 presents the definitions of the risk factors used in the study.

**Table 1:** Definitions of risk factors included in the study.

Risk factors	Definition
**Socioeconomic factors**	
Personal income	The 2021 census median individual income for Indigenous populations (AUD 540 per week) was used to dichotomise as ‘1’ = ‘average or above’, ‘2’ = ‘below average’.
Highest educational attainment	Grouped as ‘1’ = ‘Completed year 12 or above’ or ‘2’ = ‘Did not complete year 12’.
Place of residence	Grouped as ‘1’ = ‘non-remote’ or ‘2’ = ‘remote’.
**Health risk factors**	
Daily smoking	Defined as smoking any number of cigarettes daily and categorised as ‘1’ = ‘non-smoker’, or ‘2’ = ‘daily smoker’.
Excessive alcohol drinking	‘Excessive' drinking was defined as consuming over 21 units (168 g or 213 ml) of alcohol weekly, based on criteria outlined in a recent publication in Lancet Public Health and guidelines from the National Health and Medical Research Council (NHMRC).^34^^,^^35^ Grouped as ‘1’ = ‘non-excessive’ or ‘2’ = ‘excessive drinking’
Physical activity	Physical activity was defined according to the 2014 Physical Activity and Sedentary Behaviour guidelines.^36^ Categorised as ‘1’ = ‘active’ or ‘2’ = ‘inactive’.
Central obesity	Central obesity defined as a waist circumference of ≥102 cm for males and ≥88 cm for females.^37^ Grouped as ‘1’ = ‘no’ or ‘2’ = ‘yes’.
**Cultural factors**	
Affiliation to an Indigenous tribe or language group	Identify as being affiliated to a specific tribal or language group. Grouped as ‘1’ = ‘yes’ or ‘2’ = ‘no’.
Main language spoken at home	Main language spoken at home. Grouped as ‘1’ = ‘Other’ or ‘2’ = ‘English’.
Level of Indigenous knowledge	Satisfaction with the level of Indigenous knowledge one possesses and grouped as ‘1’ = ‘yes’ or ‘2’ = ‘no’.
Health service use	General use of health services at the ACCHS or any other institutions. Grouped as ‘1’ = ‘ACCHS’ or ‘2’ = ‘other’.
**Past adverse experiences**	
Discrimination (unfair treatment) in the last 12 months	Defined as having experiences of discrimination (e.g., called names, racial comments, ignored, not trusted, unfairly arrested, humiliated, having objects thrown at them) because of Indigenous backgrounds in the last 12 months. Grouped as ‘1’ = ‘no’ or ‘2’ = ‘yes’.
Removed from natural family at some point	Defined as ever been removed from natural family by the government. Grouped as ‘1’ = ‘no’ or ‘2’ = ‘yes’.
Physical harm in the last 12 months	Defined as having experiences of physically hurt or harmed by someone on purpose, including physical fights in the last 12 months. Grouped as ‘1’ = ‘no’ or ‘2’ = ‘yes’.

ACCHS, Aboriginal Community Controlled Health Services.

### Potential covariates

For this study, we considered potential covariates including age, sex (grouped as male or female), and marital status (grouped as married or unmarried).

### Statistical analysis

All data were accessed in the ABS DataLab for analysis. Frequencies and percentages were initially calculated to present an overview of the study population. All frequencies and percentages were weighted using the personal weight variable (fingerwt), and the sample size was approximated by dividing the personal weight variable by 100.

Logistic regression models were used to compute odds ratios (ORs) with 95% confidence intervals (CIs) for the modifiable risk factors associated with depression and anxiety disorder. These variables were selected based on their relevance to the outcomes and their amenability to interventions. The performance of the logistic regression models was evaluated by receiver operating characteristic (ROC) curves. Our analysis involved the computation of PAFs using Miettinen's formula. The choice of Miettinen's formula was based on its ability to produce reliable estimates, particularly in the presence of confounding, when adjusted ORs are used.^38^ PAF was calculated using the following formula:

PAF=Pc(OR−1)/OR

where, Pc is the prevalence of the modifiable risk factor among cases, and OR is the adjusted ORs.^38^^,^^39^

Given that modifiable risk factors occur simultaneously within individuals, aggregating the PAF for each specific risk factor may lead to an overestimation of their combined effects.^40^^,^^41^ We employed communality weights to correct for the overlap of risk factors among the study participants.^35^ To calculate the communalities, we initially computed the pairwise tetrachoric correlation among all potentially modifiable risk factors. Subsequently, a principal components analysis was conducted on the tetrachoric correlation matrix. The communality for each risk factor was determined by the sum of squares of the loadings in all principal components with an eigen value > 1. The weighting of each risk factor (We) was then carried out using the formula: We = 1 − communality.

Following this, a combined PAF across the potentially modifiable risk factors was calculated using the specified formula:

$$PAF\;\left(combined\right)=1-\prod_{r=1}^{R}\left(1\;-WePAFe\right)$$

where ‘e’ represents each modifiable risk factor, and ‘We’ represents the communality weight of each risk factor.

Finally, we estimated the adjusted PAF for each risk factor using the formula:

adjustedPAFe=([PAFe/∑PAFe]∗combinedPAF

The 95% CIs for PAF estimates were calculated using the standard method for 95% CIs for proportions, with the sample size being the total number of participants, consistent with a previously published study.^35^ All statistical analyses were conducted using Stata version 18.0 (StataCorp, USA) with ‘svy’ command to adjust for effect of sampling and stratification.

### Ethics approval

Ethical clearance for this study was obtained from the Charles Sturt University Human Research Ethics Committee (H23808), and the ABS provided clearance to the data analysis output files. The involvement of First Nations People in reviewing the study's concept, design, and implementation were facilitated through Regional Enterprise Development Institute (REDI.E) and Orange Aboriginal Medical Service (OAMS), aligning with the National Health and Medical Research Council (NHMRC) guideline for conducting ethical research with Aboriginal and Torres Strait Islander peoples.

### Role of the funding source

Funders had no role in study design, data collection, data analysis, interpretation or writing of the report.

## Results

### Study participants

This study included a weighted sample of 5362 individuals, with a mean age of 40.8 years (SD = ±17.2), and 51.7% were female. Of the total participants, 64.1% (3435) were not married and 38.0% (2037) lived in major cities. A total of 47.8% (2566) made less than the national income, and 48.2% (2501) did not complete Year 12. About 37.4% (2004) smoked daily, 12.1% (646) had an excessive drinking problem, 74.1% (3950) were physically inactive and 65.7% (3520) had central obesity.

Only 12.9% (689) knew that they were affiliated to an Indigenous tribe or language group, and 9.7% (523) did not speak English as the main language at home. While 54.2% (2637) were satisfied with the level of Indigenous knowledge they possessed and 33.8% (1811) utilized services provided by ACCHS. A total of 24.9% (1160) participants had experienced discrimination because of their indigenous backgrounds in the last 12 months and 6.3% (308) had experienced physical harm in the last 12 months. A total of 15.4% (710) of the participants were removed from their natural family by the government at some point (Table 2).

**Table 2:** Characteristics of the study participants.

	Male (N = 2589)	Female (N = 2772)	Total (N = 5361)
	n (%)	n (%)	n (%)
**Socioeconomic factors**			
**Age-group**			
15–24	729 (28.81)	667 (24.9)	1396 (26.8)
25–34	575 (22.7)	605 (22.6)	1180 (22.7)
35–44	397 (15.7)	440 (16.4)	836 (16.1)
45–54	378 (14.9)	437 (16.4)	816 (15.7)
55–64	270 (10.7)	306 (11.4)	576 (11.1)
65+	180 (7.1)	218 (8.1)	398 (7.6)
**Marital status**			
Married	924 (35.7)	1002 (36.2)	1926 (35.9)
Not married	1665 (64.3)	1770 (63.8)	3435 (64.1)
**Place of residence (remoteness)**			
Major cities	986 (38.1)	1051 (37.9)	2037 (38.0)
Outer regional	602 (23.2)	636 (22.9)	1238 (23.1)
Inner regional	508 (19.6)	554 (20.0)	1036 (19.8)
Remote	173 (6.7)	187 (6.8)	360 (6.7)
Very remote	320 (12.4)	343 (12.4)	663 (12.4)
**Personal income**			
Average or above	1422 (54.9)	1374 (49.6)	2796 (52.2)
Below average	1167 (45.1)	1398 (50.4)	2566 (47.8)
**Highest educational attainment**			
≥Year 12	1269 (50.4)	1421 (53.2)	2691 (51.8)
<Year 12	1251 (49.6)	1251 (46.8)	2501 (48.2)
**Health risk factors**			
**Daily smoking**			
Yes	1008 (38.9)	996 (35.9)	2004 (37.4)
No	1581 (61.1)	1776 (64.1)	3357 (62.6)
**Excessive drinking alcohol**			
Yes	482 (18.6)	164 (5.9)	646 (12.1)
No	2107 (81.4)	2608 (94.1)	4715 (87.9)
**Physical activity**			
Inactive	1844 (71.6)	2106 (76.3)	3950 (74.1)
Active	733 (28.4)	653 (23.7)	1385 (25.9)
**Central obesity**			
Yes	1432 (55.3)	2087 (75.3)	3520 (65.7)
No	1157 (44.7)	685 (24.7)	1842 (34.3)
**Cultural factors**			
**Identify as a specific tribal or language group**			
Yes	326 (12.6)	364 (13.1)	689 (12.9)
No	2263 (87.4)	2408 (86.9)	4672 (87.1)
**Main language spoken at home**			
English	2338 (90.3)	2500 (90.2)	4839 (90.3)
Non-English	251 (9.7)	272 (9.8)	523 (9.7)
**Satisfied with the level of cultural knowledge**			
Yes	1317 (57.4)	1320 (52.5)	2637 (54.2)
No	978 (42.6)	1192 (47.5)	2170 (45.1)
**Places for health service use**			
ACCHS	848 (32.8)	962 (34.7)	1811 (33.8)
Other institutions	1741 (67.2)	1810 (65.3)	3551 (66.2)
**Past adverse experiences**			
**Experienced discrimination because of indigenous background in the last 12 months**			
Yes	532 (24.2)	628 (25.5)	1160 (24.9)
No	1671 (75.8)	1829 (74.5)	3500 (75.1)
**Ever removed from natural family by the government**			
Yes	302 (13.9)	407 (16.8)	710 (15.4)
No	1878 (86.1)	2015 (83.2)	3893 (84.6)
**Experienced physical harm in last 12 months**			
Yes	149 (6.4)	159 (6.2)	308 (6.3)
No	2188 (93.6)	2405 (93.8)	4592 (93.7)
**Common mental disorders**			
**Depression**			
Yes	361 (13.9)	610 (22.0)	971 (18.1)
No	2228 (86.1)	2162 (78.0)	4391 (81.9)
**Anxiety**			
Yes	352 (13.6)	742 (26.8)	1094 (20.4)
No	2237 (86.4)	2030 (73.2)	4267 (79.6)

ACCHS, Aboriginal Community Controlled Health Services.

The prevalence of depression among Individuals aged 15 years or more was 18.1% (95% CI: 16.5, 19.7), with the rate higher among females (22.0%, 95% CI: 19.7, 24.4) than males (13.9%, 95% CI: 12.0, 16.2). Similarly, the prevalence of anxiety was 20.4% (95% CI: 18.7, 22.2), with the rate higher among females (26.8%, 95% CI: 24.2, 29.4) than males (13.6%, 95% CI: 11.5, 16.0).

### Population attributable fractions for depression

**Fig. 1:**
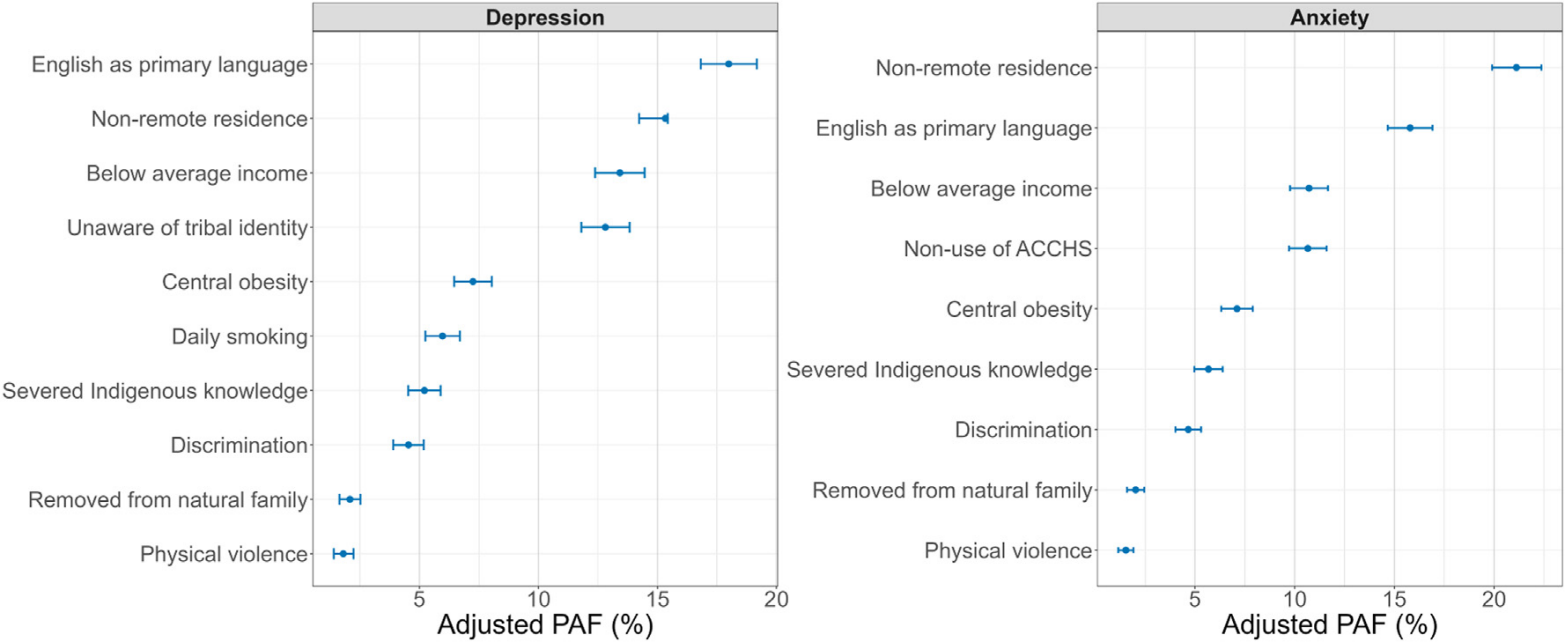
Population-attributable fractions for depression and anxiety among Aboriginal and Torres Strait Islander peoples aged ≥15 years. (ACCHS, Aboriginal Community Controlled Health Services).

The combined PAF showed that five potentially modifiable risk factors were associated with 44.5% (95% CI: 40.3, 48.8) of depression cases among Aboriginal and Torres Strait Islander peoples (Table 3, Fig. 1). These risk factors included: personal income below the national average (PAF = 13.4%; 95% CI: 12.4, 14.5), severed access to Indigenous cultural affiliations (PAF = 12.8%; 95% CI: 11.8, 13.8), central obesity (PAF = 7.2%; 95% CI: 6.4, 8.0), daily smoking (PAF = 5.9%; 95% CI: 5.2, 6.7) and severed access to Indigenous knowledge (PAF = 5.2%; 95% CI: 4.5, 5.8).

**Table 3:** Determinants and population-attributable fractions for depression among Aboriginal and Torres Strait Islander peoples aged ≥15 years (n = 4097).

	Risk factor prevalence % (95% CI)	OR (95% CI)	Unadjusted PAF %	1-Communality %	Adjusted PAF % (95% CI)
**Socioeconomic factors**					
**Personal income**					
Below average	64.8 (60.2, 69.1)	2.83 (2.15, 3.71)	41.90	52.53	13.42 (12.38, 14.46)
Average or above	35.2 (30.8, 39.7)	Ref	Ref		Ref
**Place of residence**					
Non-remote	92.6 (90.8, 94.0)	2.07 (1.49, 2.88)	47.87	71.32	15.33 (14.23, 15.43)
Remote	7.4 (5.9, 9.1)	Ref	Ref		Ref
**Health risk factors**					
**Daily smoking**					
Yes	46.9 (42.1, 51.7)	1.66 (1.28, 2.15)	18.65	59.45	5.97 (5.25, 6.70)
No	53.0 (48.2, 57.8)	Ref	Ref		Ref
**Central obesity**					
Yes	75.3 (70.9, 79.1)	1.43 (1.07, 1.92)	22.64	48.94	7.25 (6.46, 8.04)
No	24.7 (20.8, 29.0)	Ref	Ref		Ref
**Cultural factors**					
**Identify as a specific tribal or language group**					
No	92.1 (89.7, 93.9)	1.77 (1.23, 2.55)	40.01	70.90	12.81 (11.80, 13.83)
Yes	7.9 (6.1, 10.3)	Ref	Ref		Ref
**Main language spoken at home**					
English	97.7 (96.7, 98.5)	2.35 (1.35, 4.08)	56.12	72.18	18.00 (16.82, 19.18)
Non-English	2.3 (1.5, 3.4)	Ref	Ref		Ref
**Satisfied with the level of cultural knowledge**					
No	58.0 (53.0, 62.7)	1.39 (1.07, 1.80)	16.27	70.90	5.21 (4.53, 5.89)
Yes	42.0 (37.2, 46.9)	Ref	Ref		Ref
**Past adverse experiences**					
**Experienced discrimination in the last 12 months**					
Yes	35.0 (30.3, 39.8)	1.68 (1.28, 2.21)	14.17	51.08	4.54 (3.90, 5.18)
No	65.0 (60.1, 69.6)	Ref	Ref		Ref
**Ever removed from natural family**					
Yes	20.6 (17.2, 24.6)	1.46 (1.08, 1.97)	6.49	46.67	2.08 (1.64, 2.52)
No	79.3 (75.3, 82.7)	Ref	Ref		Ref
**Experienced physical harm in last 12 months**					
Yes	10.9 (8.3, 14.1)	2.07 (1.34, 3.19)	5.63	63.04	1.80 (1.40, 2.23)
No	89.1 (85.9, 91.7)	Ref	Ref		Ref

CI, Confidence interval; PAF, population attributable fraction; OR, odds ratio.

Adjusted PAF is the relative contribution of each risk factor to the overall PAF when adjusted for communality.

Model adjusted for age, sex, and marital status.

Highest educational attainment, excessive alcohol drinking, physical activity, and healthcare use were not found to be significant and thus were not presented in the table.

### Population attributable fractions for anxiety disorder

The combined PAFs showed that five potentially modifiable risk factors were associated with 38.8% (95% CI: 35.5, 42.9) of anxiety cases among Aboriginal and Torres Strait Islander peoples (Table 4, Fig. 1). These risk factors include: personal income below the national average (PAF = 10.7%; 95% CI: 9.8, 11.7), limited access to ACCHS (PAF = 10.6%; 95% CI: 9.7, 11.6), central obesity (PAF = 7.1%; 95% CI: 6.3, 7.9), severed access to Indigenous knowledge (PAF = 5.7%; 95% CI: 4.9, 6.4) and experiences of discrimination in the last 12 months (PAF = 4.7%; 95% CI: 4.0, 5.3).

**Table 4:** Determinants and population-attributable fractions for anxiety disorder among Aboriginal and Torres Strait Island peoples aged ≥15 years (n = 4097).

	Risk factor prevalence % (95% CI)	OR (95% CI)	Unadjusted PAF %	1-Communality %	Adjusted PAF % (95% CI)
**Socioeconomic factors**					
**Personal income**					
Below average	57.2 (52.3, 61.9)	2.11 (1.62, 2.75)	30.09	52.53	10.72 (9.77, 11.67)
Average or above	42.7 (38.0, 47.6)	Ref	Ref		Ref
**Place of residence**					
Non-remote	94.6 (93.2, 95.7)	2.68 (1.91, 3.75)	59.30	71.32	21.12 (19.90, 22.37)
Remote	5.4 (4.2, 6.7)	Ref	Ref		Ref
**Health risk factors**					
**Central obesity**					
Yes	75.4 (71.0, 79.2)	1.36 (1.05, 1.84)	19.95	48.94	7.11 (6.32, 7.90)
No	24.6 (20.7, 28.9)	Ref	Ref		Ref
**Cultural factors**					
**Main language spoken at home**					
English	98.6 (97.7, 99.1)	2.00 (1.03, 3.90)	49.30	70.90	15.79 (14.67, 16.91)
Non-English	1.3 (0.8, 2.2)	Ref	Ref		Ref
**Satisfied with the level of cultural knowledge**					
No	61.0 (56.2, 65.6)	1.41 (1.09, 1.83)	17.74	72.18	5.68 (4.97, 6.39)
Yes	38.9 (34.3, 43.7)	Ref	Ref		Ref
**Places for health service use**					
Other institutions	78.4 (74.7, 81.7)	1.74 (1.29, 2.33)	33.3	70.90	10.66 (9.72, 11.60)
ACCHS	21.5 (18.2, 25.2)	Ref	Ref		Ref
**Past adverse experiences**					
**Experienced discrimination in the last 12 months**					
Yes	34.5 (30.1, 39.2)	1.73 (1.32, 2.27)	14.56	51.08	4.66 (4.02, 5.31)
No	65.4 (60.7, 69.8)	Ref	Ref		Ref
**Ever removed from natural family**					
Yes	19.5 (16.2, 23.3)	1.48 (1.10, 1.99)	6.32	46.67	2.02 (1.59, 2.45)
No	80.4 (76.6, 83.7)	Ref	Ref		Ref
**Experienced physical harm in last 12 months**					
Yes	9.2 (7.2, 13.1)	2.08 (1.32, 3.20)	4.78	63.04	1.53 (1.15, 1.91)
No	90.8 (86.8, 92.7)	Ref	Ref		Ref

ACCHS, Aboriginal Community Controlled Health Services; OR, odds ratio; PAF, population attributable fraction.

Adjusted PAF is the relative contribution of each risk factor to the overall PAF when adjusted for communality.

Model adjusted for age, sex, and marital status.

Highest educational attainment, daily smoking, excessive alcohol drinking, physical activity, and healthcare use were not found to be significant and thus were not presented in the table.

## Discussion

This is the first study to compute PAFs for key modifiable risk factors for depression and anxiety disorder among Aboriginal and Torres Strait Islander peoples aged ≥15 years. Our findings revealed that five potentially modifiable risk factors were associated with 45% of depression cases. These risk factors included: lower personal income, severed access to Indigenous knowledge and cultural affiliations, central obesity, and daily smoking. Similarly, five potentially modifiable risk factors were associated with 39% of anxiety cases. The risk factors included: lower personal income, limited access to ACCHS, central obesity, severed access to Indigenous knowledge, and experiences of discrimination in the last 12 months.

The risk factors investigated in this study hold broader implications for preventing and managing depression and anxiety disorder in Aboriginal and Torres Strait Islander peoples. The computation of PAF for depression and anxiety disorder provides an opportunity to prioritize the most important modifiable risk factors, allowing for the optimization of available resources to address the growing mental health needs within Aboriginal and Torres Strait Islander communities. Based on our PAF estimates, programs and policies can be more effective in preventing and managing depression and anxiety disorder if they integrate strategies to engage Indigenous communities and promote access to indigenous cultural assets and leadership. Increasing access to healthy diet and active lifestyle remain critical component as well.

In addition, it is imperative to address broader social determinants of well-being, including systemic racism (in healthcare, education, employment, and justice systems), forced removal, and incarceration of Indigenous peoples.^7^^,^^12^^,^^19^^,^^42^ Comprehensive policy reforms are necessary to address these issues. These reforms include reforming the justice system,^43^ implementing anti-racism training across public services^44^ and implementing economic empowerment initiatives (e.g., improving employment opportunities, supporting Indigenous businesses, and ensuring equitable access to education).^45^ Additionally, acknowledging traditional ownership of land and waters (the Native Title Act 1993) for Aboriginal and Torres Strait Islander peoples and promoting awareness of Indigenous histories and cultures among non-Indigenous populations are crucial for fostering a more inclusive society.^22^^,^^46^

A considerable portion of depression and anxiety disorder were attributable to past traumatic experiences, including experiences of racism and discrimination, and the removal of individuals from their natural families during childhood, consistent with previous studies.^7^^,^^47^ While integrative, personalized, stepped-care approaches co-designed and implemented by Indigenous-led institutions are crucial for healing co-occurring (intergenerational) trauma and depression-anxiety among specific sub-populations, it is equally important to address the root causes.^25^^,^^48^ Implementing upstream measures may include adopting the United Nations Declaration on the Rights of Indigenous Peoples, providing constitutional recognition of Aboriginal and Torres Strait Islanders, enacting anti-racist legislation to address institutional racism, reforming education to include Indigenous perspectives, and improving economic opportunities through policies that support land rights and sovereignty.^49^ These strategies could be essential components of a comprehensive approach to closing the mental health gap and enhancing the overall well-being of Aboriginal and Torres Strait Islander populations.

In the present study, health risk factors such as smoking and central obesity significantly contributed to depression and anxiety disorder among Aboriginal and Torres Strait Islander peoples. The burden of central obesity is escalating in remote, poor Aboriginal and Torres Strait Islander communities due to the existence of ‘food deserts’, characterized by inequities in the availability, price, promotion, and quality of healthy food sources.^50^ Depression and anxiety disorder, along with other chronic conditions, constitute a substantial component of the overall Indigenous health gap, necessitating the implementation of coordinated and comprehensive approaches to create equitable healthy environments.^4^^,^^51^ Programs to promote healthy diet and lifestyles are more inclusive and effective when co-designed with Indigenous communities and linked to Indigenous knowledge and culture.^52^ These programs may include providing health information through traditional Yarning and Dadirri methods, promoting traditional land-based physical activities (e.g., hunting, gathering, and participation in other customs and traditions),^52^ and supporting the production of and access to healthy, affordable foods (e.g., startup loans and governance support).^53^ Furthermore, the association between mental disorders and central obesity could provide a useful guide in planning personalized interventions (e.g., social prescribing^54^) within community and primary care settings.

The role of ACCHS is particularly important in providing culturally sensitive, mental health care, as noted in the present study. Furthermore, cultural adaptation of generic mental health services is necessary to meet the cultural requirements of specific Indigenous groups.^19^^,^^20^^,^^22^^,^^55^^,^^56^ Shortages of Indigenous mental health workers (e.g., psychologists, specialists), non-availability of mental health services in ACCHS, lack of coordination between ACCHS and mainstream mental health services, and limited funding are potential implementation barriers that current Indigenous mental health programs are facing.^21^^,^^22^^,^^25^ State and national governments should further increase spending in enhancing the capacity of Indigenous-led organizations to provide mental health and support services.

Program approaches that are based on Indigenous cultural values and leadership may also mitigate the effects of structural disadvantages (e.g., poverty and poor living conditions, systemic racism, intergenerational trauma) on the existing mental health gap in Aboriginal and Torres Strait Islander communities.^19^, ^20^, ^21^, ^22^ Therefore, existing strength-based approaches should ensure Aboriginal and Torres Strait Islander leaders' ownership of policy decisions, as well as the design and implementation of programs at all levels, including regular reporting and involvement in evaluating outcomes. Self-determining Indigenous solutions that prioritize the autonomy and agency of Indigenous communities in areas such as healthcare, justice, education, child protection and economic development are imperative for increasing resilience and empowerment, as well as for facilitating culturally tailored approaches.^57^ State and national governments should increase investment in implementing Indigenous mental health and wellbeing programs to foster Indigenous self-determination and leadership.

### Strengths and limitations of the study

This study has strengths. The nationally representative sample allowed us to examine the determinants and PAFs for key modifiable risk factors for depression and anxiety disorder. We found area under the curve (AUC) values of 0.7481 for the logistic regression model predicting anxiety and 0.7352 for the model predicting depression, indicating that both models performed well in predicting their respective outcomes (Supplementary file 1).

This study also had limitations. First, the use of cross-sectional data presents difficulties in establishing a temporal relationship between covariates and the outcome variable. Second, the outcome variables and most of the explanatory variables were measured based on self-reported questionnaires and which could be a source of recall bias. Third, the outcome variables were the diagnosis by a health professional and thus, the PAFs' are likely underestimating impacts. For instance, given there is inequitable access to mental health services and healthcare, it is likely this greatly underestimates population prevalence of mental health distress among Aboriginal and Torres Strait Islander populations, as most will not access treatment or assessment. Fourth, our selection of cultural variables was based on their relevance with subject under investigation, and the completeness of the data available for analysis. We did not use any specific cultural frameworks (such as Anangu cultural domains—Tjukurpa, Kanyi, Walta, and Ngura)^42^ because the 2018–19 NATSIHS survey did not collect data on all these cultural domains.

Fifth, some people might not have disclosed information about physical harm and discrimination to an interviewer due to lack of privacy during the interview process. Last, PAF estimates rely on particular assumptions, involving causality, the independence of modifiable risk factors, and consistent associations over time.^58^ However, these assumptions might prove unrealistic due to the intricate interplay of socio-economic, cultural, healthcare, and behavioural factors associated with depression and anxiety disorder. Despite this complexity, PAFs offer a straightforward and intuitive metric that can supplement other methodologies in pinpointing modifiable risk factors suitable for policy intervention.

### Conclusion

Lower personal income, central obesity, and constrained access to cultural assets were associated with 45% of depression cases and 39% of anxiety cases among Aboriginal and Torres Strait Islander peoples in this study. Specifically, severed access to Indigenous knowledge and cultural affiliations with limited access to ACCHS increased inequity. To reduce the burden of depression and anxiety disorder in this population, addressing socio-economic and cultural harms that constrain healthy connections to people/kin, their rights, languages, land, and healthy food sources should be a priority. State and national governments should increase investment in reducing systemic racism and implementing Indigenous mental health and wellbeing programs to foster Indigenous self-determination and leadership. This approach will promote mental health and wellbeing for Aboriginal and Torres Strait Islanders and will contribute to the broader goal of closing the gap.

## Contributors

ST and KYH conceptualised the study idea, obtained and analysed the data, interpreted the results. ST drafted the original manuscript. KYA, SG, AEA, MMH, PG, SM, FHA, JN and AGR critically revised the manuscript for intellectual content. KYA, MMH, PG, JN and AGR contributed to the conception of the research idea, and interpretation and critically revised the manuscript. ST, KYA and MMH had full access to the raw data and KYA and MMH accessed and verified the data. PG and JN, who are Indigenous community leaders and the CEOs of two Aboriginal-led health institutions, were involved in the study conception, variable selection, and interpretation of the findings and both revised the manuscript, focusing on improving cultural sensitivity, methodological appropriateness, respect for cultural values, and ensuring ethical considerations. AGR provided supervision for the research. All authors read and approved the final submission of the study for publication.

## Data sharing statement

Weighted prevalence estimates from the Australian Bureau of Statistics (ABS) surveys used in the current study are available on the ABS website. Aggregated weighted data from these surveys are also hosted by the ABS on their TableBuilder online platform, where users can perform limited analyses (e.g., calculating prevalence by age groups). The ABS also stores the deidentified unit record-level data that underly the national surveys on their DataLab online platform. Users can perform more detailed analyses, such as generating variables not available publicly, and all results are vetted by ABS staff before release. Access to ABS TableBuilder and the ABS DataLab requires formal approval processes.

## Declaration of interests

The authors declare no conflict of interests.
