# Supplementary material for: Population attributable fractions of depression and anxiety among Aboriginal and Torres Strait Islander peoples: a population-based study
Source: Lancet Reg Health West Pac. 2024 Sep 20;52:101203. doi: 10.1016/j.lanwpc.2024.101203 (PMC11458540; doi:10.1016/j.lanwpc.2024.101203)
Supplement: Supplementary file 1 [file mmc1.docx]

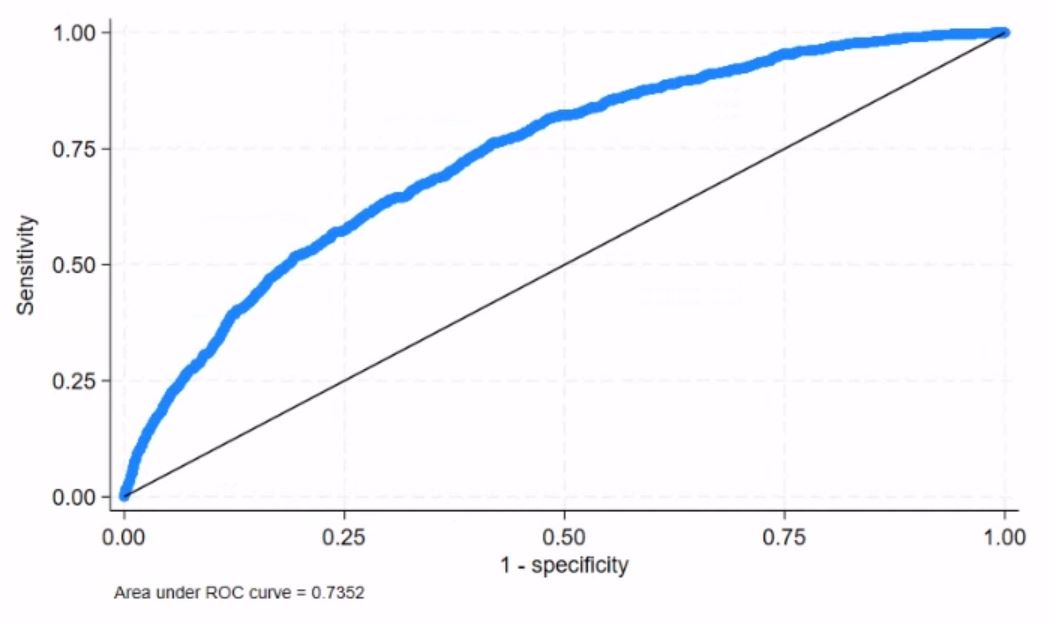


Figure 1. Receiver operating characteristic (ROC) curve of logistic regression model predicting depression in Aboriginal and Torres Strait Islander peoples aged 15 years and older


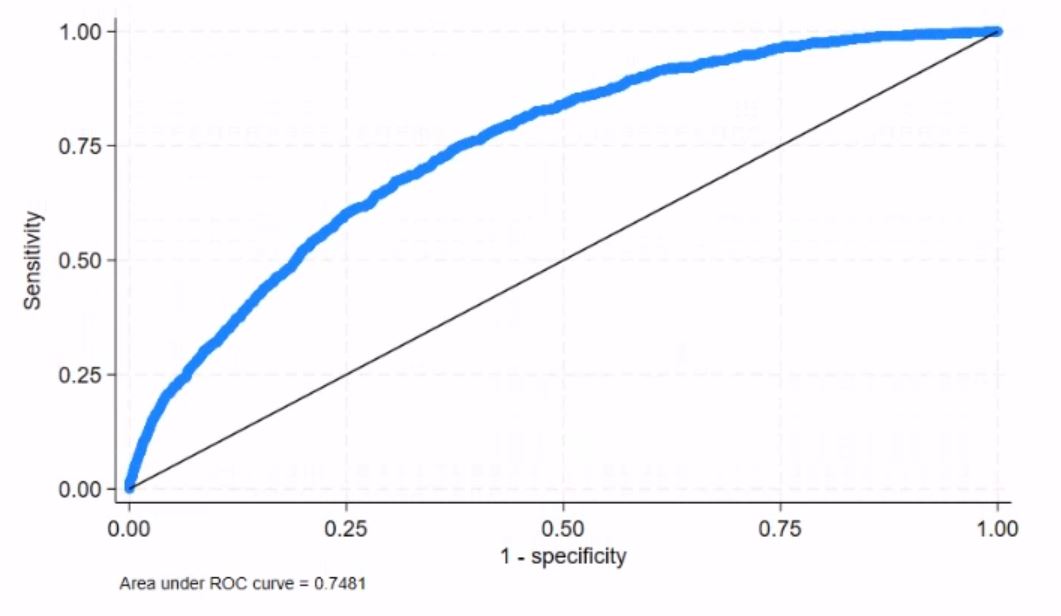


Figure 2. Receiver operating characteristic (ROC) curve of logistic regression model predicting anxiety disorder in Aboriginal and Torres Strait Islander peoples aged 15 years and older
